# Association between triglyceride-glucose index and risk of end-stage renal disease in patients with type 2 diabetes mellitus and chronic kidney disease

**DOI:** 10.3389/fendo.2023.1150980

**Published:** 2023-04-20

**Authors:** Yue-Ming Gao, Wei-Jia Chen, Zhen-Ling Deng, Zhi Shang, Yue Wang

**Affiliations:** ^1^ Department of Nephrology, Peking University Third Hospital, Beijing, China; ^2^ Department of Cardiology and Institute of Vascular Medicine, Peking University Third Hospital, Beijing, China

**Keywords:** triglyceride-glucose index, insulin resistance, type 2 diabetes mellitus, chronic kidney disease, end-stage renal disease

## Abstract

**Aims:**

It has been suggested that the triglyceride-glucose (TyG) index is a novel and reliable surrogate marker of insulin resistance (IR). However, its relationship with the risk of end-stage renal disease (ESRD) in patients with type 2 diabetes mellitus (T2DM) and chronic kidney disease (CKD) remains uncertain. Accordingly, we sought to examine the relationship between the TyG index and ESRD risk in patients with T2DM and CKD.

**Methods:**

From January 2013 to December 2021, 1,936 patients with T2DM and CKD hospitalized at Peking University Third Hospital (Beijing, China) were enrolled into the study. The formula for calculating the TyG index was ln[fasting triglyceride (mg/dL) × fasting blood glucose (mg/dL)/2]. ESRD was defined as an estimated glomerular filtration rate of less than 15 mL/min/1.73 m^2^ or the commencement of dialysis or renal transplantation. The relationship between the TyG index and ESRD risk was analyzed using Cox proportional hazard regression.

**Results:**

105 (5.42%) participants developed ESRD over a mean follow-up of 41 months. The unadjusted analysis revealed a 1.50-fold (95% confidence interval [CI] 1.17-1.93; *P* = 0.001) increased risk for ESRD per one unit rise in the TyG index, and the positive association remained stable in the fully adjusted model (hazard ratio, 1.49; 95% CI, 1.12-1.99; *P* = 0.006). Analysis using restricted cubic spline revealed a significant positive association between the TyG index and ESRD risk. In addition, Kaplan-Meier analysis revealed significant risk stratification with a TyG index cutoff value of 9.5 (*P* = 0.003).

**Conclusion:**

In individuals with T2DM and CKD, a significant and positive association was shown between an elevated TyG index and the risk of ESRD. This conclusion provides evidence for the clinical importance of the TyG index for evaluating renal function decline in individuals with T2DM and CKD.

## Introduction

Type 2 diabetes mellitus (T2DM) has become a worldwide epidemic that poses a significant threat to human health (1). As a major microvascular complication of T2DM, chronic kidney disease (CKD) was estimated to affect 25–40% of all patients with T2DM (2). In addition, approximately 30% of patients with T2DM will ultimately progress to end-stage renal disease (ESRD) (3). Currently, T2DM is the primary cause of end-stage renal disease (ESRD) in developed countries (4) and the second cause of ESRD in China (5). T2DM-related ESRD not only leads to a reduction in survival rate (6) and health-related quality of life (7) but also brings a heavy economic burden to patients and society (8).

Insulin resistance (IR), characterized as the inability of cells to react to insulin action, is a prominent feature of T2DM and is linked to a wide range of clinical conditions, including cognitive impairment (9), non-alcoholic fatty liver disease (10), cardiovascular disease (CVD) (11), and CKD (12). Accumulated data indicate that IR has an impact on numerous facets of kidney function, such as kidney hemodynamics, podocyte viability, and the function of renal tubules (13). Additionally, IR promotes glomerular hypertrophy and renal interstitial fibrosis, which result in hypertension and albuminuria and may speed up the progression of CKD (12). As a result, accessing IR may be important for predicting the occurrence and progression of T2DM-related renal complications.

Recently, the triglyceride-glucose (TyG) index, generated from fasting triglyceride (TG) and fasting blood glucose (FBG), has a strong association with IR assessed by the hyperinsulinemic-euglycemic clamp (HIEC) test and the homeostasis model assessment of IR (HOMA-IR) (14–16). Thus, the TyG index has been suggested as a simple and promising surrogate indicator for IR. Previous clinical data have shown associations of the TyG index with the occurrence of T2DM (17–19) and diabetic macrovascular (20, 21) or microvascular complications (22–24). Additionally, in a prospective cohort study conducted by Low et al. (25), a higher TyG index was found to be independently correlated with CKD progression in patients with T2DM, which was partially mediated by pigment epithelium-derived factor. Furthermore, in a recent study conducted by Fritz et al. (26), the TyG index was demonstrated to be positively associated with the risk of ESRD and mediates approximately 50% of the entire association between body mass index (BMI) and ESRD in the general population. However, studies investigating the association between the TyG index and ESRD risk in patients with T2DM and CKD are scarce.

Thus, the purpose of the present study was to analyze the relationship between the TyG index and ESRD risk in individuals with T2DM and CKD.

## Materials and methods

### Study population

**Figure 1:**
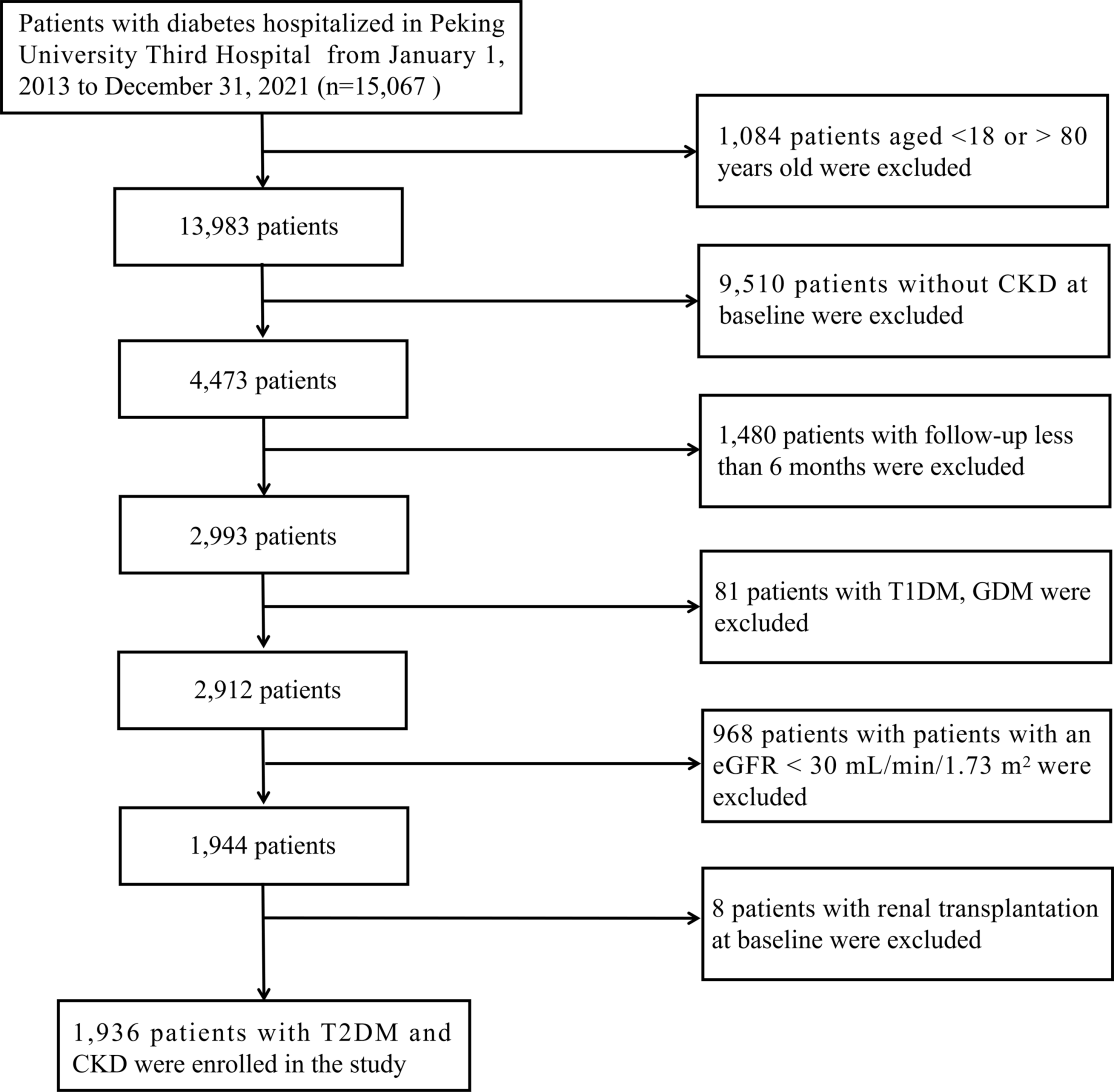
Enrollment flowchart for the research population.

This retrospective cohort study included 15,067 patients with diabetes who were hospitalized at Peking University Third Hospital (Beijing, China) between January 2013 and December 2021. The following were the inclusion criteria: 1) between the ages of 18 and 80; 2) a confirmed diagnosis of CKD; 3) a follow-up for more than six months; CKD was confirmed as a sustained estimated glomerular filtration rate (eGFR) < 60 mL/min/1.73 m^2^ and/or the presence of albuminuria (≥ 1+ in routine urine test) in at least two out of three consecutive measurements during a six-month period at baseline. The following were the exclusion criteria: 1) patients with a confirmed diagnosis of type 1 diabetes mellitus (T1DM) or gestational diabetes mellitus (GDM); 2) patients with a baseline eGFR of less than 30 mL/min/1.73 m^2^; 3) patients received renal transplantation at baseline. Finally, 1,936 eligible patients were included in our study. **Figure 1** depicts the enrollment flowchart for the research population. The study was carried out in accordance with the Declaration of Helsinki and approved by the Ethics Committee of Peking University Third Hospital (IRB00006761-M207365).

### Data collection and definitions

Data for each patient were gathered from the digital patient records system of Peking University Third Hospital. Clinical characteristics, including age, age of onset of DM, duration of diabetes, sex, body mass index (BMI), systolic blood pressure (SBP), diastolic blood pressure (DBP), mean arterial pressure (MAP), the medical history of hyperlipidemia, heart failure (HF), hypertension, stroke, anemia, smoking status, and the treatment of insulin and renin-angiotensin-aldosterone system (RAAS) inhibitors were collected. After an 8- to 12- h overnight fast, venous blood samples of each patient were obtained on the second morning of hospitalization. Laboratory parameters, white blood cells (WBC), hemoglobin (HGB), serum albumin (ALB), serum uric acid, serum creatinine (Scr), eGFR, blood urea nitrogen (BUN), triglyceride (TG), high-density lipoprotein cholesterol (HDL-C), low-density lipoprotein cholesterol (LDL-C), total cholesterol (TC), fasting blood glucose (FBG), glycated hemoglobin (HbA1c), and urinary protein level (0–4+), were collected at baseline.

The BMI, determined as body weight measured in kilograms divided by height in meters squared and was classified according to Chinese criteria for Chinese patients (27), including normal weight (18.5–24 kg/m^2)^, overweight (24–28 kg/m^2^), and obesity (≥ 28 kg/m^2^). Self-reported smoking status was classified into former, current, and nonsmoker. Individuals who have given up smoking for a minimum of six months were defined as former smokers. Current smokers were defined as those who had smoked regularly over the previous six months. Nonsmokers were individuals who had never smoked throughout their lifetime (28). Hyperlipidemia was defined as the current use of lipid-lowering medications, the presence of any self-reported history, or the fulfillment of any of the following conditions: TG ≥ 2.26 mmol/Lor serum TC ≥ 6.22 mmol/L or LDL-C ≥ 4.14 mmol/L or HDL-C < 1.04 mmol/L (29). Hypertension was defined as an elevated SBP (≥ 140 mmHg) or DBP (≥ 90 mmHg), or a self-reported history of hypertension (30). According to World Health Organization criteria, anemia is determined as HGB levels below 120 g/L in women and 130 g/L in men (31). MAP was calculated by adding one-third of pulse pressure (SBP-DBP) to DBP. The Chronic Kidney Disease Epidemiology Collaboration (CKD-EPI) equation was used to determine the eGFR (32). When calculating the TyG index, the formula was ln[fasting TG (mg/dL) × FBG (mg/dL)/2] (33).

### Covariates

We adopted the sensitivity analysis method in the multivariate Cox regression analysis. Potential covariates were pre-specified according to our clinical experience and the current literature on predictors for renal function decline in T2DM patients (34). These covariates included basic clinical characteristics (sex, age of DM onset, duration of diabetes, smoking status, MAP, and BMI), complications or comorbidities (hyperlipidemia, HF, and anemia), medication history (lipid-lowering drugs and insulin), and laboratory examinations (eGFR, urinary protein, LDL-C, and HbA1c). Each covariate was determined at the time of the first hospitalization.

### Study outcome

The occurrence of ESRD, including an eGFR of less than 15 mL/min/1.73 m^2^ or the commencement of dialysis (hemodialysis or peritoneal dialysis) or renal transplantation, was considered as the study outcome. The initial hospitalization was used to determine the start of observation. Follow-up ended at the diagnosis of ESRD or the time of the latest hospitalization or clinic visit.

### Statistical analysis

Categorical variables were shown as percentages and compared using the chi-squared test or Fisher’s exact test between two groups. Depending on the variable distribution, continuous variables were displayed as mean ± standard deviation or median [interquartile range (IQR)] and compared using the independent sample *t*-test or the Mann-Whitney U test. There were some missing data in several variables (the percentage of missing data < 10%). Missing data were considered to be missing randomly and imputed *via* random forests-based missing data imputation (R package missForest) (35). Multivariable-adjusted Cox proportional hazards regression was employed to estimate the adjusted hazard ratios (HR) and 95% confidence intervals (CI). In order to investigate the relationship between the TyG index and ESRD risk, the TyG index was handled as a continuous variable and analyzed using a restricted cubic spline (RCS) with three knots. Time to the event in each group of TyG index (decided by the cut-off value) at baseline was presented by the Kaplan-Meier curve and the significance was determined using the log-rank test. Furthermore, we have conducted further analysis stratified by age (≥ 60 or < 60 years), age of onset of DM (≥ 58 or < 58 years), DM duration (≥ 12 or < 12 months), sex (male or female), anemia (yes or no), MAP (≥ 100 or < 100 mmHg), hyperlipidemia (yes or no), insulin treatment (yes or no), BMI (≥ 24 or < 24 kg/m^2^), HF (yes or no), stroke (yes or no), and RAAS inhibitor treatment (yes or no) to explore the differences among these subgroups. Analyses were performed using R software, version 4.1.1. R software (version 4.1.1) was used to conduct the statistical analysis. All P-values were two-sided, with < 0.05 deemed statistically significant.

## Results

### Baseline clinical characteristics

Among the 1,936 participants, the median (IQR) age was 67 (59, 74) years at baseline, and 1,395 (71.1%) were male. The median TyG index was 9.16 (IQR 8.74–9.64). **Table 1** describes the baseline clinical features of the research population according to the study outcome. Participants with outcome were slightly younger, more frequently female, had a longer duration of diabetes, had an earlier age of onset of DM, and had a higher prevalence of hypertension, anemia, RAAS inhibitors and insulin treatment. They also showed higher levels of SBP, DBP, MAP, Scr, BUN, LDL-C, TC, TG, HbA1c, and urinary protein, and had lower levels of HGB, serum ALB, and eGFR. Notably, participants with outcomes had a significantly higher TyG index compared to patients without outcomes (9.34 [8.76, 9.99] vs. 9.16 [8.74, 9.62], *P*=0.012).

**Table 1:** Baseline clinical characteristics of the study population.

Characteristics	Overall (n=1,936)	Patients with outcome (n=105)	Patients without outcome (n=1,831)	*P*-value
Demographics				
Age (years)	67 (59,74)	62 (55,71)	67 (59,74)	<0.001
Age of DM onset (years)	66 (58, 73)	61 (53, 70)	66 (58, 73)	<0.001
Age of DM onset category (n, %)				<0.001
< 58 years	450 (23.2%)	42 (40%)	408 (22.3%)	
≥ 58 years	1486 (76.8%)	63 (60%)	1423 (77.7%)	
Sex (male, %)	1,395 (72.1%)	62 (59.0%)	1,333 (72.8%)	0.003
Duration of diabetes (months)	8.5 (1, 15)	12 (7, 20)	8 (1, 15)	<0.001
Duration of diabetes category (n, %)				<0.001
< 12 months	1,306 (67.5%)	50 (47.6%)	1,256 (68.6%)	
≥ 12 months	630 (32.5%)	55 (52.4%)	575 (31.4%)	
BMI (kg/m^2^)	25.6 (23.6, 27.7)	25.2 (22.9, 27.6)	25.6 (23.7, 27.7)	0.332
BMI category (n, %)				0.243
< 24	563 (29.1%)	38 (36.2%)	525 (28.7%)	
24 – 28	948 (49.0%)	45 (42.9%)	903 (49.3%)	
≥ 28	425 (22.0%)	22 (21.0%)	403 (22.0%)	
Smoking status (n, %)				0.605
Nonsmoker	990 (51.1%)	50 (47.6%)	940 (51.3%)	
Former smoker	771 (39.8%)	43 (50%)	728 (39.8%)	
Current smoker	175 (9.0%)	12 (11.4%)	163 (8.9%)	
Complications or comorbidities				
Hyperlipidemia (n, %)	963 (49.7%)	49 (46.7%)	914 (49.9%)	0.584
HF (n, %)	222 (11.5%)	17 (16.2%)	205 (11.2%)	0.160
Hypertension (n, %)	1,509 (77.9%)	92 (87.6%)	1,417 (77.4%)	0.019
Stroke (n, %)	336 (17.4%)	23 (21.9%)	313 (17.1%)	0.257
Anemia (n, %)	655 (33.8%)	61 (58.1%)	594 (32.4%)	<0.001
Treatment				
Insulin (n, %)	652 (33.7%)	51 (48.6%)	601 (32.8%)	0.001
RAAS inhibitors (n, %)	1060 (54.8%)	68 (64.8%)	992 (54.2%)	0.044
Physical and laboratory findings				
SBP (mmHg)	135 (124, 149)	144 (125, 159)	135 (124, 148)	<0.001
DBP (mmHg)	78 (70, 84)	80 (75, 90)	78 (70, 84)	<0.001
MAP (mmHg)	97 (90, 104)	101 (94, 113)	96 (90, 103)	<0.001
MAP category (n, %)				<0.001
≥ 100 mmHg	744 (38.4%)	58 (55.2%)	686 (37.5%)	
< 100 mmHg	1,192 (61.6%)	47 (44.8%)	1,145 (62.5%)	
WBC (×10^9^/L)	6.79 (5.65, 8.19)	6.95 (5.77, 8.58)	6.77 (5.63, 8.14)	0.224
HGB (g/L)	134 (121, 145)	120 (107, 136)	134 (122, 146)	<0.001
Serum ALB (g/L)	39.8 (36.7, 42.8)	36.7 (32.6, 40.2)	39.9 (37.0, 42.9)	<0.001
Scr (μmol/L)	98 (90, 110)	112 (99, 128)	97 (90, 109)	<0.001
BUN (mmol/L)	6.8 (5.6, 8.3)	8.5 (6.7, 10.4)	6.7 (5.5, 8.2)	<0.001
Serum uric acid (μmol/L)	379 (322, 443)	387 (330, 464)	378 (321, 442)	0.138
eGFR (mL/min/1.73 m2)	52.2 (45.0, 57.6)	45.5 (37.8, 53.6)	52.5 (45.4, 57.7)	<0.001
eGFR category (n, %)				<0.001
≥ 90	72 (3.7%)	3 (2.9%)	69 (3.8%)	
60 – 89	157 (8.1%)	11 (10.5%)	146 (8.0%)	
45 – 59	1,223 (63.2%)	41 (39%)	1,182 (64.6%)	
30 – 44^b^	484 (25.0%)	50 (47.6%)	434 (23.7%)	<0.001
LDL-C (mmol/L)	2.44 (1.87, 3.12)	2.74 (2.05, 3.48)	2.43 (1.86, 3.10)	0.005
LDL-C category (n, %)				0.007
≥ 2.6	833 (43.0%)	59 (56.2%)	744 (40.6%)	
< 2.6	1,103 (57.0%)	46 (43.8%)	1.087 (59.4%)	
HDL-C (mmol/L)	0.96 (0.82, 1.13)	0.96 (0.81, 1.17)	0.96 (0.82, 1.12)	0.798
TC (mmol/L)	4.12 (3.44, 5.03)	4.96 (3.99, 5.74)	4.09 (3.41, 4.98)	<0.001
TG (mmol/L)	1.64 (1.19, 2.35)	1.99 (1.46, 2.79)	1.63 (1.18, 2.33)	0.002
FBG (mmol/L)	7.0 (5.8, 8.9)	7.2 (5.5, 9.7)	7.0 (5.9, 8.9)	0.641
HbA1c (%)	7.6 (6.7, 8.9)	8.1 (7.0, 9.9)	7.5 (6.7, 8.8)	0.003
HbA1c category (n, %)				0.086
≥ 7%	1,299 (67.1%)	79 (75.2%)	1,220 (66.6%)	
< 7%	637 (32.9%)	26 (24.8%)	611 (33.4%)	
TyG index	9.16 (8.74, 9.64)	9.34 (8.76, 9.99)	9.16 (8.74, 9.62)	0.012
Urinary protein grade (n, %)				<0.001
0 – ±	1,364 (70.5%)	22 (21.0%)	1,342 (73.3%)	
1+ – 2+^a^	349 (18.0%)	32 (30.5%)	317 (17.3%)	
3+ – 4+ ^a,b^	223 (11.5%)	51 (48.6%)	172 (9.4%)	

a represented that there was a significant difference (P < 0.05) between the current category and the first category of the variable.

b represented that there was a significant difference (P < 0.05) between the current category and the second category of the variable.

### Association of the baseline TyG index with the risk of ESRD

As shown in **Table 2**, in the univariate Cox regression, each one-unit increment of TyG index was significantly associated with an increased ESRD risk (HR = 1.50, 95%CI 1.17-1.93, P = 0.001). In this investigation, four multivariate adjustment models were developed (**Table 2**), in which model 1 adjusted for sex, age of DM onset, duration of diabetes, hyperlipidemia, and treatment of lipid-lowering drugs at baseline. The results indicated that the TyG index was significantly positively correlated with ESRD risk (HR = 1.34, 95% CI 1.02-1.76, P = 0.037). Model 2 further adjusted for smoking status, insulin treatment, and HF on the basis of model 1, and the results revealed that the positive association between the TyG index and ESRD risk remained stable (HR = 1.35, 95% CI 1.03-1.78, P = 0.032). Model 3 further adjusted for baseline eGFR, MAP, and BMI on the basis of model 2, and the association between TyG index and ESRD risk remained stable (HR = 1.36, 95% CI 1.03-1.79, P = 0.029). Finally, after further adjusting for anemia, urinary protein, LDL, and HbA1c on the basis of model 3, the positive correlation between the TyG index and ESRD remained significant (HR = 1.49, 95% CI 1.12-1.99, P = 0.006). The details of the covariates were presented in **Supplementary Figure 1** and **Supplementary Table 1**. In summary, the TyG index was an independent predictor for ESRD in patients with T2DM and CKD.

**Table 2:** HR (95% CI) for risk of ESRD according to the TyG index at baseline.

Model	HR	95% CI	*P*-value
Unadjusted model	1.50	1.17-1.93	0.001
Model 1	1.34	1.02-1.76	0.037
Model 2	1.35	1.03-1.78	0.032
Model 3	1.36	1.03-1.79	0.029
Model 4	1.49	1.12-1.99	0.006

Model 1 adjusted for sex, age of DM onset, duration of diabetes, hyperlipidemia, and treatment of lipid-lowering drugs at baseline; model 2 adjusted for variables in model 1 plus smoking status, insulin treatment, and HF at baseline; model 3 adjusted for variables in model 2 plus eGFR, MAP, and BMI at baseline; model 4 adjusted for variables in model 3 plus anemia, urinary protein, LDL-C, and HbA1c at baseline.

**Figure 2:**
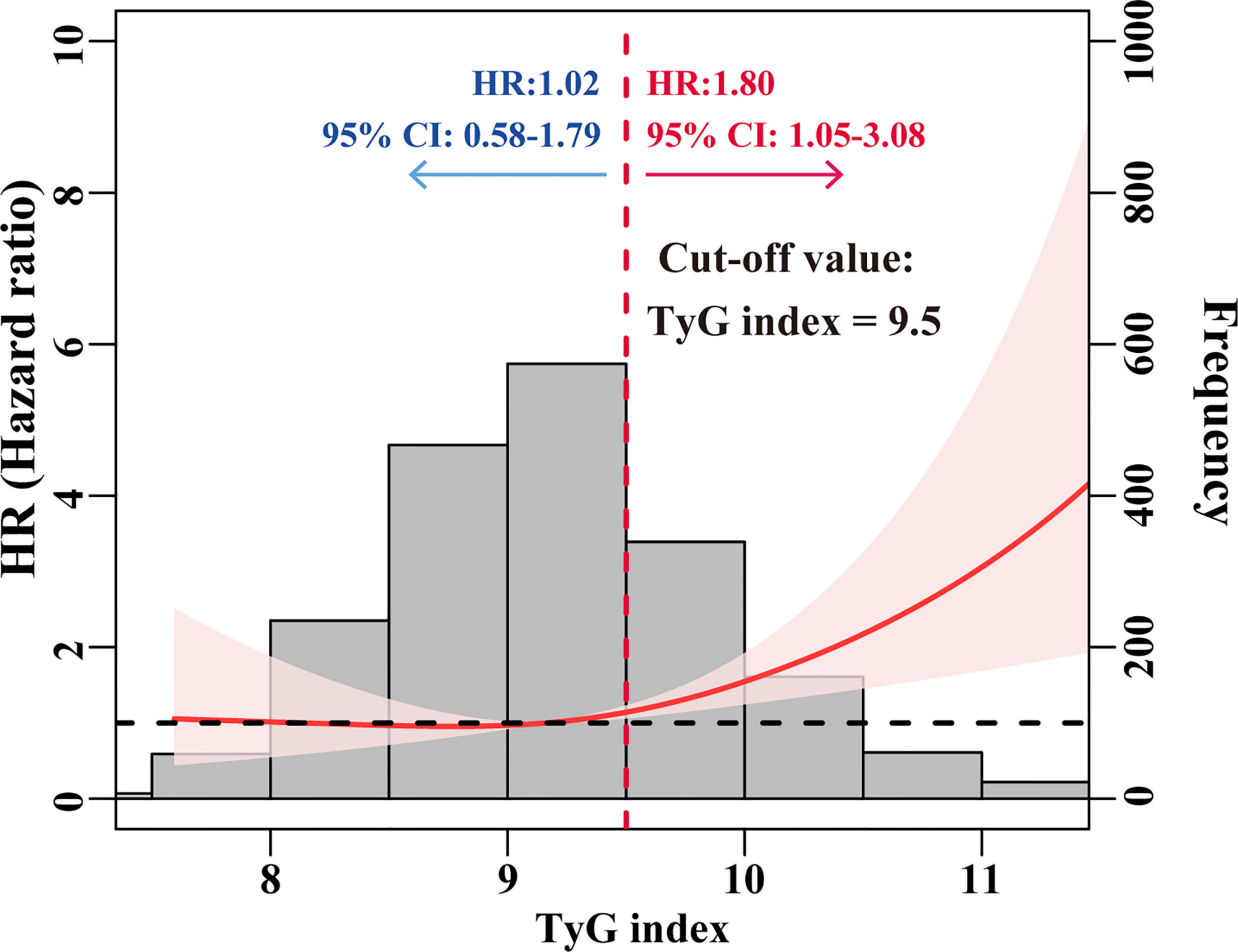
HRs for the risk of ESRD by baseline TyG index. The red solid line represented unadjusted HRs of the baseline TyG index across the whole range. The black dot line represents the reference line when HR = 1. The frequency distribution of the baseline TyG index is shown by histograms.

As shown in **Figure 2**, we utilized the RCS curve to model and depict the relationship between baseline TyG index and the risk of ESRD in patients with T2DM and CKD, which demonstrated that the patients with TyG index ≥ 9.5 had a relatively higher risk for ESRD. So we chose a cut-off value of 9.5 and categorized the baseline TyG index as follows: TyG index < 9.5 and ≥ 9.5. The risk of ESRD did not increase with TyG index in patients with TyG index < 9.5, while it was significantly increased with TyG index in patients with TyG index ≥ 9.5 (HR = 1.80; 95%CI: 1.05-3.08).

**Figure 3:**
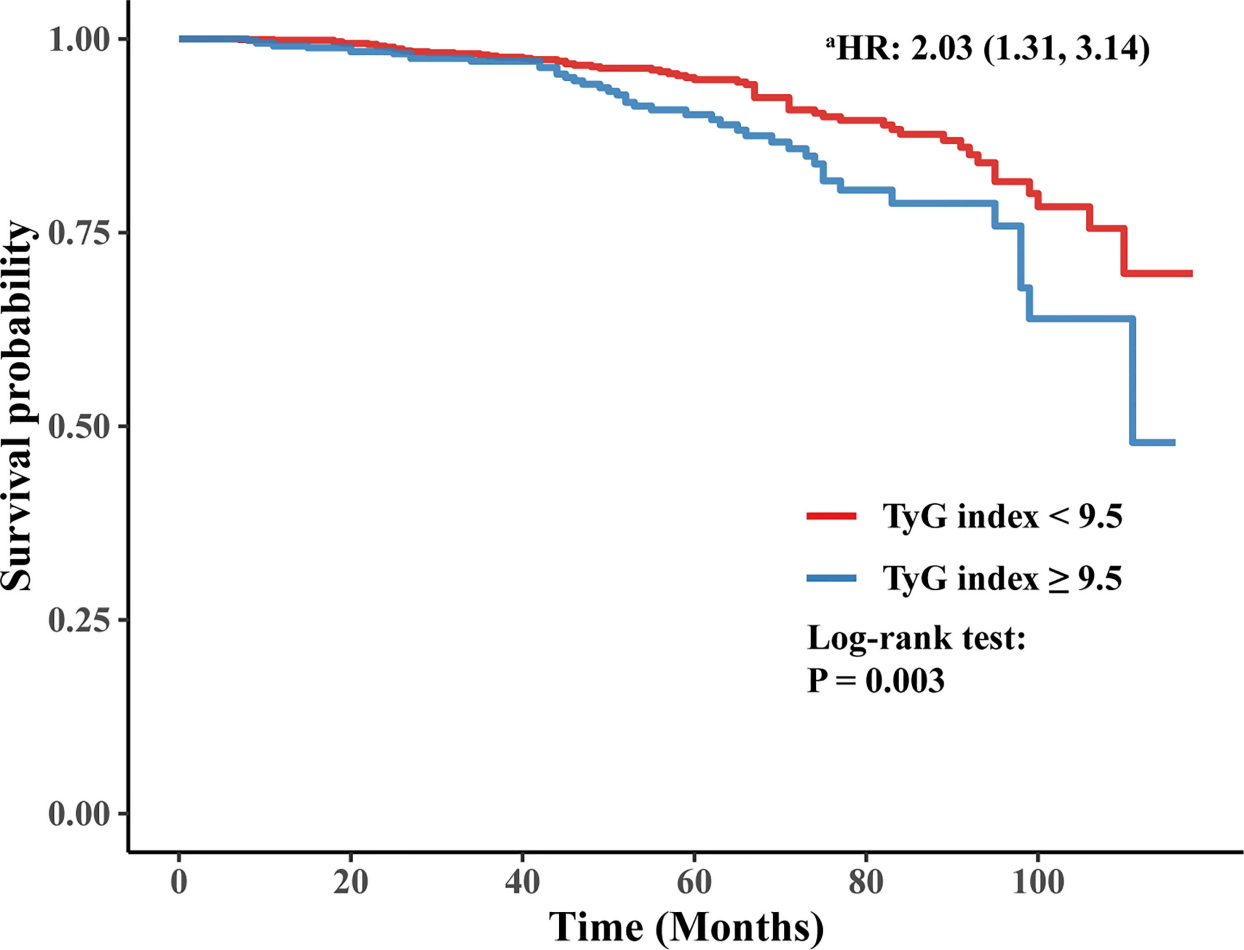
Kaplan-Meier curve of time to incident ESRD. ^a^: adjusting for sex, age of DM onset, duration of diabetes, hyperlipidemia, treatment of lipid-lowering drugs, smoking status, insulin treatment, HF, eGFR, MAP, BMI, anemia, urinary protein, LDL, and HbA1c.

The Kaplan-Meier curve also showed that participants with a higher baseline TyG index (≥ 9.5) were more likely to develop ESRD than those with a baseline TyG index < 9.5 (log-rank test, *P* = 0.003). Furthermore, after adjusting for sex, age of DM onset, duration of diabetes, hyperlipidemia, treatment of lipid-lowering drugs, smoking status, insulin treatment, HF, eGFR, MAP, BMI, anemia, urinary protein, LDL, and HbA1c, the HR for ESRD in participants with a baseline TyG index ≥ 9.5 was 2.03 (95% CI 1.31-3.14) compared to participants with a baseline TyG index < 9.5. The Kaplan-Meier curve is shown in **Figure 3**.

### Subgroup analysis

**Figure 4:**
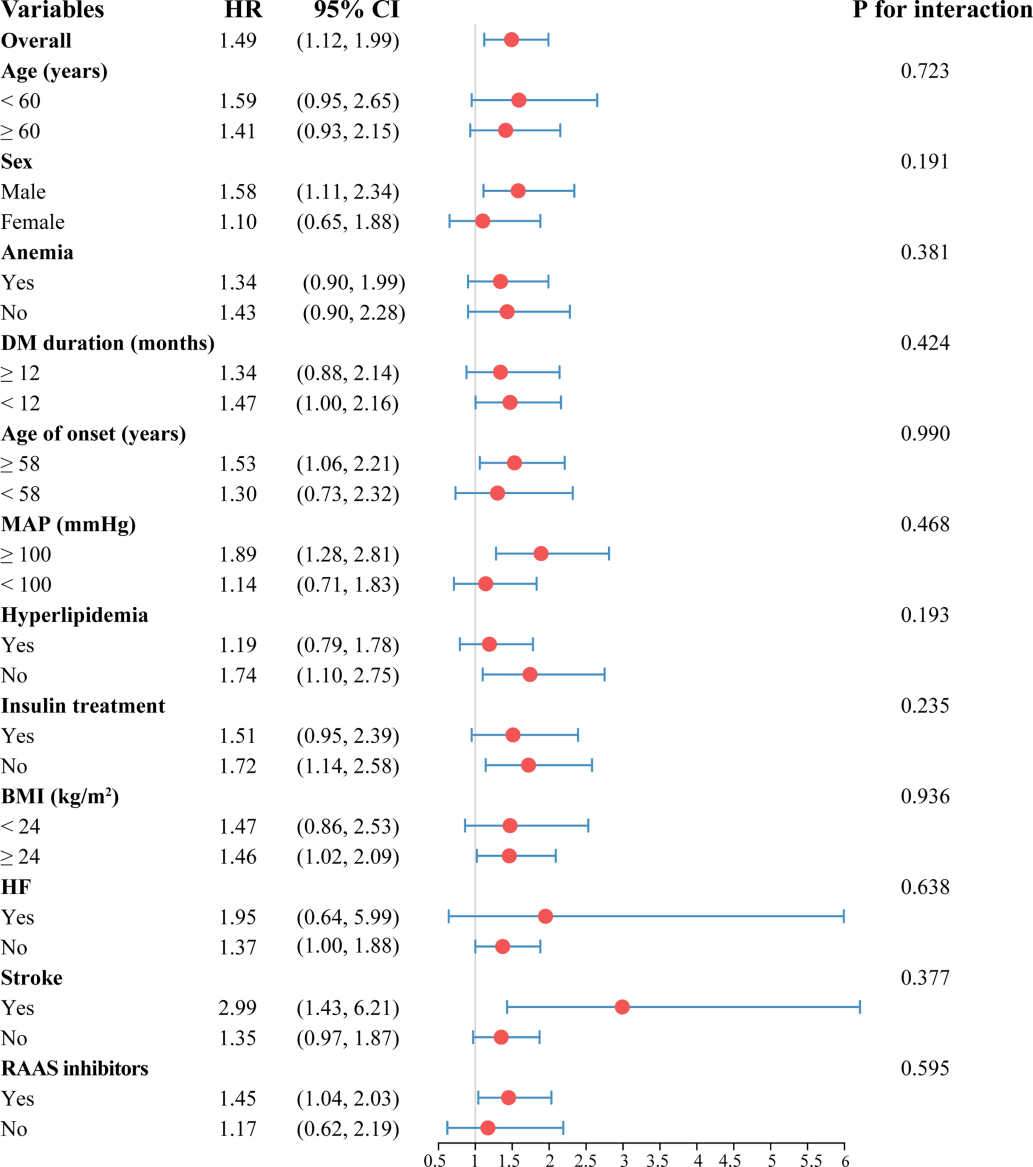
Subgroup analysis of the association between baseline TyG index and ESRD risk.

To further explore the impact of additional risk factors on the correlation of the TyG index with the risk of ESRD, subgroup analyses were conducted in accordance with the following stratification variables: age (≥ 60 or < 60 years), age of onset of DM (≥ 58 or < 58 years), DM duration (≥ 12 or < 12 months), sex (male or female), anemia (yes or no), MAP (≥ 100 or < 100 mmHg), hyperlipidemia (yes or no), insulin treatment (yes or no), BMI (≥ 24 or < 24 kg/m^2^), HF (yes or no), stroke (yes or no), and RAAS inhibitor treatment (yes or no). **Figure 4** compiles the outcomes of subgroup analysis and interactions. Across all subgroups, the TyG index had a statistically significant positive relationship with the risk of ESRD. The association between the TyG index and the risk of ESRD remained consistent among all subgroups (*P* for interaction > 0.05).

## Discussion

In this single-center retrospective cohort study, the baseline TyG index was found to be positively associated with the risk of future ESRD in patients with T2DM and CKD. After adjusting for well-known and suspected risk factors, including sex, age of DM onset, duration of diabetes, smoking status, insulin treatment, hyperlipidemia, baseline eGFR, MAP, BMI, anemia, urinary protein, and HbA1c, the positive association between the TyG index and ESRD remained stable, indicating that baseline TyG index was an independent predictor for ESRD in patients with T2DM and CKD. Specifically, participants with a baseline TyG index ≥ 9.5 had a significantly high risk of future ESRD and poor prognosis. Kaplan-Meier analysis also showed significant risk stratification when using 9.5 as a cut-off value of the baseline TyG index.

IR appears in a very early stage of CKD in patients with or without diabetes and worsens as renal functions decline (36). The etiology of IR in CKD is complex and multifactorial, including unhealthy lifestyles, chronic inflammation, oxidative stress, uremic toxins, metabolic acidosis, anemia, vitamin D deficiency, and disturbed gut microbiome (36–38). IR accelerates the development and progression of CKD *via* various mechanisms, including worsening renal hemodynamics, affecting podocyte viability, promoting glomerular mesangial expansion and renal tubular interstitial fibrosis (12, 13).

Conventional methods for directly measuring IR are complicated, invasive, and relatively expensive. Thus, accessible and inexpensive tests to determine IR have been developed and compared to the existing gold standard, the HIEC test (14, 15, 39). The HOMA-IR, which provides an estimate for IR from FBG and insulin serum concentrations, is a well-validated and commonly used method to measure IR. However, a plasma insulin test is not yet accessible in all laboratories and is not routinely measured in clinical practice (16). The TyG index, derived from simple and less costly biochemical measurements, has been shown to have a strong correlation with the HIEC test (14, 15) and HOMA-IR (16). Therefore, in our retrospective cohort study, we adopted the TyG index as a surrogate marker of IR since it was more accessible in routine clinical practice.

A major finding of our study was that an elevated TyG index, a surrogate marker of IR, is significantly associated with a higher risk of ESRD in individuals with T2DM and CKD. It was suggested that the TyG index appeared to be associated with incident CKD (21–24, 40) and CKD progression (25, 41) in patients with or without diabetes. Recently, Fritz et al. (26) discovered that in the general population, the TyG index appeared to play a significant mediating role between BMI and ESRD, and tended to be correlated with ESKD risk. In our study, even after adjusting for BMI, the positive association between the TyG index and future ESRD remained stable in patients with T2DM and CKD, suggesting monitoring the TyG index may be taken into consideration in these patients to identify and take the necessary precautions to prevent future ESRD.

This study has several limitations. First, this is a retrospective cohort study, which may suffer from bias in report and selection. Second, our study is a single-center study, with a modest sample size. Future studies with a bigger sample size and a longer follow-up time are required to verify the link between the TyG index and ESRD in individuals with T2DM and CKD.

## Conclusion

In patients with T2DM and CKD, an elevated TyG index was significantly associated with a higher risk of ESRD. This finding supports the clinical use of the TyG index, a simple and practical surrogate marker of IR, for assessing renal function deterioration in patients with T2DM and CKD.

## Data availability statement

The raw data supporting the conclusions of this article will be made available by the authors, without undue reservation.

## Ethics statement

The studies involving human participants were reviewed and approved by The Ethics Committee of Peking University Third Hospital (IRB00006761-M207365). Written informed consent for participation was not required for this study in accordance with the national legislation and the institutional requirements.

## Author contributions

ZS and YW designed the study. Y-MG and W-JC collected the data. Y-MG, W-JC and ZS analyzed the data. Y-MG and W-JC interpreted the result. Y-MG and W-JC prepared the first draft of the manuscript. YW and Z-LD revised the manuscript. All authors contributed to the article and approved the submitted version.

## References

[1] Sun H Saeedi P Karuranga S Pinkepank M Ogurtsova K Duncan BB. IDF diabetes atlas: global, regional and country-level diabetes prevalence estimates for 2021 and projections for 2045. Diabetes Res Clin Pract (2022) 183:109119. doi: 10.1016/j.diabres.2021.109119 34879977 PMC11057359

[2] Pugliese G Penno G Natali A Barutta F Di Paolo S Reboldi G. Diabetic kidney disease: new clinical and therapeutic issues. joint position statement of the Italian diabetes society and the Italian society of nephrology on "The natural history of diabetic kidney disease and treatment of hyperglycemia in patients with type 2 diabetes and impaired renal function". J Nephrol (2020) 33(1):9–35. doi: 10.1007/s40620-019-00650-x 31576500 PMC7007429

[3] Bakris GL. Update on reducing the development of diabetic kidney disease and cardiovascular death in diabetes. Kidney Int Suppl (2011) (2018) 8(1):1. doi: 10.1016/j.kisu.2017.10.002 30675432 PMC6336218

[4] Saran R Robinson B Abbott KC Bragg-Gresham J Chen X Gipson D. US Renal data system 2019 annual data report: epidemiology of kidney disease in the united states. Am J Kidney Dis (2020) 75(1 Suppl 1):A6–7. doi: 10.1053/j.ajkd.2019.09.003 31704083

[5] Liu ZH. Nephrology in china. Nat Rev Nephrol (2013) 9(9):523–8. doi: 10.1038/nrneph.2013.146 23877587

[6] González-Pérez A Saez M Vizcaya D Lind M Garcia Rodriguez L. Incidence and risk factors for mortality and end-stage renal disease in people with type 2 diabetes and diabetic kidney disease: a population-based cohort study in the UK. BMJ Open Diabetes Res Care (2021) 9(1):e002146. doi: 10.1136/bmjdrc-2021-002146 PMC8491294 34607828

[7] Chen JY Wan EYF Choi EPH Chan AKC Chan KHY Tsang JPY. The health-related quality of life of Chinese patients on hemodialysis and peritoneal dialysis. Patient (2017) 10(6):799–808. doi: 10.1007/s40271-017-0256-6 28589314

[8] Chen HY Kuo S Su PF Wu JS Ou HT. Health care costs associated with macrovascular, microvascular, and metabolic complications of type 2 diabetes across time: estimates from a population-based cohort of more than 0.8 million individuals with up to 15 years of follow-up. Diabetes Care (2020) 43(8):1732–40. doi: 10.2337/dc20-0072 PMC7372047 32444454

[9] Arnold SE Arvanitakis Z Macauley-Rambach SL Koenig AM Wang HY Ahima RS. Brain insulin resistance in type 2 diabetes and Alzheimer disease: concepts and conundrums. Nat Rev Neurol (2018) 14(3):168–81. doi: 10.1038/nrneurol.2017.185 PMC6098968 29377010

[10] Della Pepa G Vetrani C Lombardi G Bozzetto L Annuzzi G Rivellese AA. Isocaloric dietary changes and non-alcoholic fatty liver disease in high cardiometabolic risk individuals. Nutrients (2017) 9(10):1065. doi: 10.3390/nu9101065 28954437 PMC5691682

[11] Hong SH Choi KM. Sarcopenic obesity, insulin resistance, and their implications in cardiovascular and metabolic consequences. Int J Mol Sci (2020) 21(2):494. doi: 10.3390/ijms21020494 31941015 PMC7013734

[12] Whaley-Connell A Sowers JR. Insulin resistance in kidney disease: is there a distinct role separate from that of diabetes or obesity? Cardiorenal Med (2017) 8(1):41–9. doi: 10.1159/000479801 PMC5757598 29344025

[13] Artunc F Schleicher E Weigert C Fritsche A Stefan N Häring HU. The impact of insulin resistance on the kidney and vasculature. Nat Rev Nephrol (2016) 12(12):721–37. doi: 10.1038/nrneph.2016.145 27748389

[14] Guerrero-Romero F Simental-Mendía LE González-Ortiz M Martínez-Abundis E Ramos-Zavala MG Hernández-González SO. The product of triglycerides and glucose, a simple measure of insulin sensitivity. comparison with the euglycemic-hyperinsulinemic clamp. J Clin Endocrinol Metab (2010) 95(7):3347–51. doi: 10.1210/jc.2010-0288 20484475

[15] Mohd Nor NS Lee S Bacha F Tfayli H Arslanian S. Triglyceride glucose index as a surrogate measure of insulin sensitivity in obese adolescents with normoglycemia, prediabetes, and type 2 diabetes mellitus: comparison with the hyperinsulinemic-euglycemic clamp. Pediatr Diabetes (2016) 17(6):458–65. doi: 10.1111/pedi.12303 26251318

[16] Kang B Yang Y Lee EY Yang HK Kim HS Lim SY. Triglycerides/glucose index is a useful surrogate marker of insulin resistance among adolescents. Int J Obes (Lond) (2017) 41(5):789–92. doi: 10.1038/ijo.2017.14 28104918

[17] Brahimaj A Rivadeneira F Muka T Sijbrands EJG Franco OH Dehghan A. Novel metabolic indices and incident type 2 diabetes among women and men: the Rotterdam study. Diabetologia (2019) 62(9):1581–90. doi: 10.1007/s00125-019-4921-2 PMC6677703 31183505

[18] Zhang M Wang B Liu Y Sun X Luo X Wang C. Cumulative increased risk of incident type 2 diabetes mellitus with increasing triglyceride glucose index in normal-weight people: the rural Chinese cohort study. Cardiovasc Diabetol (2017) 16(1):30. doi: 10.1186/s12933-017-0514-x 28249577 PMC5333419

[19] Park B Lee HS Lee YJ. Triglyceride glucose (TyG) index as a predictor of incident type 2 diabetes among nonobese adults: a 12-year longitudinal study of the Korean genome and epidemiology study cohort. Transl Res (2021) 228:42–51. doi: 10.1016/j.trsl.2020.08.003 32827706

[20] Su WY Chen SC Huang YT Huang JC Wu PY Hsu WH. Comparison of the effects of fasting glucose, hemoglobin A1c, and triglyceride-glucose index on cardiovascular events in type 2 diabetes mellitus. Nutrients (2019) 11(11):2838. doi: 10.3390/nu11112838 31752391 PMC6893677

[21] Pan Y Zhong S Zhou K Tian Z Chen F Liu Z. Association between diabetes complications and the triglyceride-glucose index in hospitalized patients with type 2 diabetes. J Diabetes Res (2021) 2021:8757996. doi: 10.1155/2021/8757996 34671683 PMC8523276

[22] Srinivasan S Singh P Kulothungan V Sharma T Raman R. Relationship between triglyceride glucose index, retinopathy and nephropathy in type 2 diabetes. Endocrinol Diabetes Metab (2020) 4(1):e00151. doi: 10.1002/edm2.151 33532603 PMC7831221

[23] Liu L Xia R Song X Zhang B He W Zhou X. Association between the triglyceride-glucose index and diabetic nephropathy in patients with type 2 diabetes: a cross-sectional study. J Diabetes Investig (2021) 12(4):557–65. doi: 10.1111/jdi.13371 PMC8015837 33319507

[24] Ou YL Lee MY Lin IT Wen WL Hsu WH Chen SC. Obesity-related indices are associated with albuminuria and advanced kidney disease in type 2 diabetes mellitus. Ren Fail (2021) 43(1):1250–8. doi: 10.1080/0886022X.2021.1969247 PMC8409948 34461808

[25] Low S Pek S Moh A Ang K Khoo J Shao YM. Triglyceride-glucose index is prospectively associated with chronic kidney disease progression in type 2 diabetes - mediation by pigment epithelium-derived factor. Diabetes Vasc Dis Res (2022) 19(4):14791641221113784. doi: 10.1177/14791641221113784 PMC9364218 35938490

[26] Fritz J Brozek W Concin H Nagel G Kerschbaum J Lhotta K. The triglyceride-glucose index and obesity-related risk of end-stage kidney disease in Austrian adults. JAMA Netw Open (2021) 4(3):e212612. doi: 10.1001/jamanetworkopen.2021.2612 33787913 PMC8013829

[27] Zhou BF. Effect of body mass index on all-cause mortality and incidence of cardiovascular diseases–report for meta-analysis of prospective studies open optimal cut-off points of body mass index in Chinese adults. BioMed Environ Sci (2002) 15(3):245–52. 12500665

[28] Barry SA Tammemagi MC Penek S Kassan EC Dorfman CS Riley TL. Predictors of adverse smoking outcomes in the prostate, lung, colorectal and ovarian cancer screening trial. J Natl Cancer Inst (2012) 104(21):1647–59. doi: 10.1093/jnci/djs398 PMC3490843 23104210

[29] Liu X Yu S Mao Z Li Y Zhang H Yang K. Dyslipidemia prevalence, awareness, treatment, control, and risk factors in Chinese rural population: the henan rural cohort study. Lipids Health Dis (2018) 17(1):119. doi: 10.1186/s12944-018-0768-7 29788966 PMC5964901

[30] Zheng R Mao Y. Triglyceride and glucose (TyG) index as a predictor of incident hypertension: a 9-year longitudinal population-based study. Lipids Health Dis (2017) 16(1):175. doi: 10.1186/s12944-017-0562-y 28903774 PMC5598027

[31] WHO. Haemoglobin concentrations for the diagnosis of anaemia and assessment of severity. Geneva: World Health Organization (2011). Available at: https://www.who.int/health-topics/anaemia#tab=tab_1 Accessed21 November 2022.

[32] Levey AS Stevens LA Schmid CH Zhang YL Castro AF 3rd Feldman HI. A new equation to estimate glomerular filtration rate. Ann Intern Med (2009) 150(9):604–12. doi: 10.7326/0003-4819-150-9-200905050-00006 PMC2763564 19414839

[33] Simental-Mendía LE Rodríguez-Morán M Guerrero-Romero F. The product of fasting glucose and triglycerides as surrogate for identifying insulin resistance in apparently healthy subjects. Metab Syndr Relat Disord (2008) 6(4):299–304. doi: 10.1089/met.2008.0034 19067533

[34] Stekhoven DJ Bühlmann P. MissForest–non-parametric missing value imputation for mixed-type data. Bioinformatics (2012) 28(1):112–8. doi: 10.1093/bioinformatics/btr597 22039212

[35] Xu H Carrero JJ. Insulin resistance in chronic kidney disease. Nephrol (Carlton) (2017) 22 (Suppl 4):31–4. doi: 10.1111/nep.13147 29155496

[36] Radcliffe NJ Seah JM Clarke M MacIsaac RJ Jerums G Ekinci EI. Clinical predictive factors in diabetic kidney disease progression. J Diabetes Investig (2017) 8(1):6–18. doi: 10.1111/jdi.12533 PMC5217935 27181363

[37] Dave N Wu J Thomas S. Chronic kidney disease-induced insulin resistance: current state of the field. Curr Diabetes Rep (2018) 18(7):44. doi: 10.1007/s11892-018-1010-8 29884917

[38] Nakashima A Kato K Ohkido I Yokoo T. Role and treatment of insulin resistance in patients with chronic kidney disease: a review. Nutrients (2021) 13(12):4349. doi: 10.3390/nu13124349 34959901 PMC8707041

[39] Trikudanathan S Raji A Chamarthi B Seely EW Simonson DC. Comparison of insulin sensitivity measures in south asians. Metabolism (2013) 62(10):1448–54. doi: 10.1016/j.metabol.2013.05.016 PMC3889665 23906497

[40] Shi Y Hu L Li M Zhou W Wang T Zhu L. Association between the surrogate markers of insulin resistance and chronic kidney disease in Chinese hypertensive patients. Front Med (Lausanne) (2022) 9:831648. doi: 10.3389/fmed.2022.831648 35198578 PMC8859105

[41] Qin A Tan J Wang S Dong L Jiang Z Yang D. Triglyceride-glucose index may predict renal survival in patients with IgA nephropathy. J Clin Med (2022) 11(17):5176. doi: 10.3390/jcm11175176 36079108 PMC9456599

