# Supplementary material for: Association between triglyceride-glucose index and risk of end-stage renal disease in patients with type 2 diabetes mellitus and chronic kidney disease
Source: Front Endocrinol (Lausanne). 2023 Apr 20;14:1150980. doi: 10.3389/fendo.2023.1150980 (PMC10157287; doi:10.3389/fendo.2023.1150980)
Supplement: Supplementary file 1 [file DataSheet_1.docx]

Supplementary Material

Association between triglyceride-glucose index and risk of end-stage renal disease in patients with type 2 diabetes mellitus and chronic kidney disease

Yue-Ming Gao^1†^, Wei-Jia Chen^2†^, Zhen-Ling Deng ^1^, Zhi Shang ^2^* and Yue Wang ^1^*

^†^ These authors contributed equally to this work and share first authorship

*** Correspondence:**Zhi Shang
1911110441@bjmu.edu.cn

Yue Wang

bjwangyue@sina.com

# Supplementary Figures and Tables

## Supplementary Figures


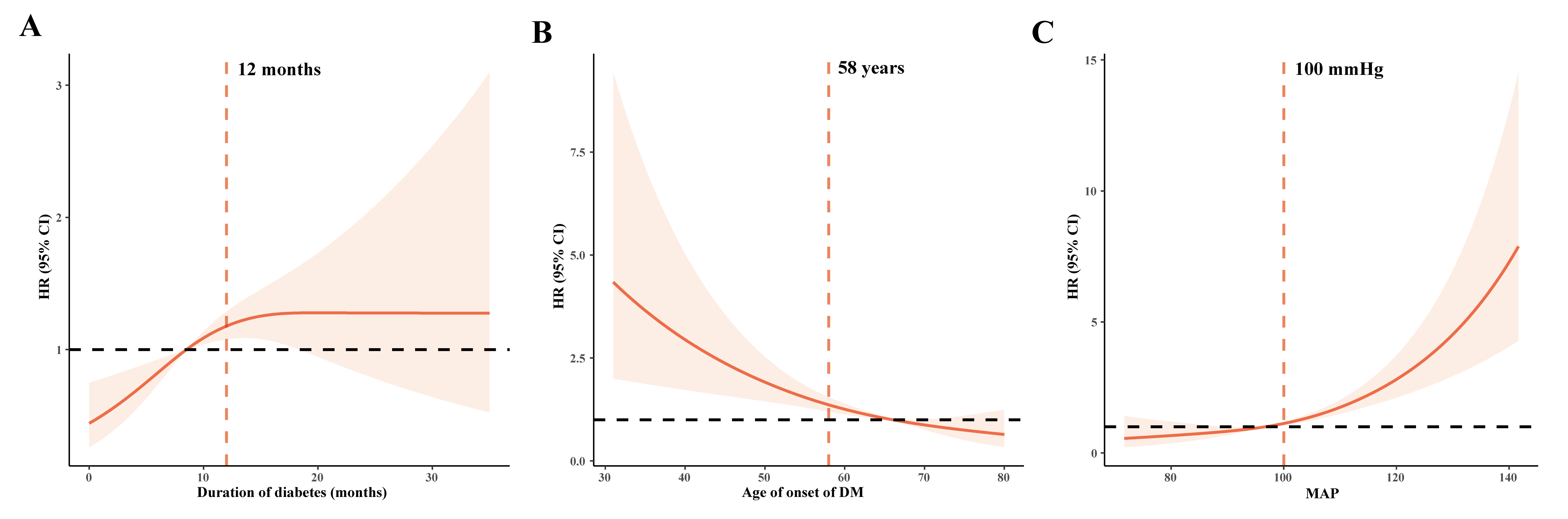


**Supplementary Figure 1.** HRs for the risk of ESRD by continuous covariates. Abbreviations: HR: hazard ratio; CI: confidence interval; DM: diabetes mellitus; MAP: mean arterial pressure.

## Supplementary Tables

**Supplementary Table 1.** Covariates adjusted in different models

|  | HR (95% CI) | | | |
| --- | --- | --- | --- | --- |
|  | Model 1 | Model 2 | Model 3 | Model 4 |
| **TyG index** | 1.34  (1.02-1.76) | 1.35  (1.03-1.78) | 1.36  (1.03-1.79) | 1.49  (1.12-1.99) |
| **Sex** |  |  |  |  |
| Male vs. female | 0.53  (0.35-0.8) | 0.40  (0.25-0.65) | 1.36  (1.03-1.79) | 1.49  (1.12-1.99) |
| **Usage of lipid lowering drugs** |  |  |  |  |
| Yes vs. no | 0.65  (0.41-1.03) | 0.53  (0.33-0.86) | 0.65  (0.4-1.06) | 0.86  (0.52-1.44) |
| **Hyperlipidemia** |  |  |  |  |
| Yes vs. no | 0.76  (0.51-1.13) | 0.77  (0.52-1.15) | 0.71  (0.47-1.07) | 0.90  (0.59-1.36) |
| **Age of onset of DM (years)** |  |  |  |  |
| ≥ 58 vs. < 58 | 0.37  (0.24-0.57) | 0.36  (0.23-0.56) | 0.34  (0.21-0.56) | 0.71  (0.43-1.18) |
| **DM duration (months)** |  |  |  |  |
| ≥ 12 vs. < 12 | 2.46  (1.64-3.70) | 2.28  (1.49-3.5) | 2.17  (1.39-3.38) | 2.33  (1.5-3.62) |
| **Insulin treatment** |  |  |  |  |
| Yes vs. no |  | 1.48  (0.98-2.23) | 1.31  (0.85-2.01) | 1.22  (0.78-1.92) |
| **Smoking status** |  |  |  |  |
| Former smoker vs. nonsmoker |  | 1.74  (1.08-2.78) | 2.35  (1.41-3.92) | 2.27  (1.34-3.83) |
| Current smoker vs. Nonsmoker |  | 1.86  (0.91-3.8) | 1.82  (0.88-3.77) | 1.82  (0.86-3.86) |
| **HF** |  |  |  |  |
| Yes vs. no |  | 2.57  (1.48-4.44) | 2.00  (1.14-3.49) | 1.77  (0.98-3.17) |
| **eGFR (mL/min/1.73m^2^)** |  |  |  |  |
| 60 – 89 vs. ≥ 90 |  |  | 5.18  (1.31-20.47) | 4.04  (1.00-16.31) |
| 45 – 59 vs. ≥ 90 |  |  | 2.83  (0.77-10.39) | 5.8  (1.56-21.63) |
| 30 – 44 vs. ≥ 90 |  |  | 8.13  (2.23-29.7) | 10.00  (2.69-37.26) |
| **MAP (mmHg)** |  |  |  |  |
| ≥ 100 vs. < 100 |  |  | 1.64  (1.08-2.49) | 1.39  (0.89-2.17) |
| **BMI (kg/m^2^)** |  |  |  |  |
| 24 –28 vs. < 24 |  |  | 0.54  (0.34-0.84) | 0.69  (0.44-1.09) |
| ≥ 28 vs. < 24 |  |  | 0.44  (0.25-0.75) | 0.54  (0.30-0.95) |
| **HbA1c (%)** |  |  |  |  |
| ≥ 7 vs. < 7 |  |  |  | 0.70  (0.42-1.18) |
| **Anemia** |  |  |  |  |
| Yes vs. no |  |  |  | 2.22  (1.41-3.47) |
| **LDL (mmol/L)** |  |  |  |  |
| ≥ 2.6 vs. < 2.6 |  |  |  | 1.50  (0.97-2.32) |
| **Urinary protein** |  |  |  |  |
| 1+ – 2+ vs. 0 –± |  |  |  | 5.96  (3.35-10.60) |
| 3+ – 4+ vs. 0 –± |  |  |  | 11.80  (6.67-20.87) |

Abbreviations: HR: hazard ratio; TyG index: triglyceride-glucose index; DM: diabetes mellitus; HF: heart failure; eGFR: estimated glomerular filtration rate; MAP: mean arterial pressure; BMI: body mass index; HbA1c: glycated hemoglobin; LDL-C: low-density lipoprotein cholesterol.
